# Respiratory Morbidity and Lung Function Analysis During the First 36 Months of Life in Infants With Bronchopulmonary Dysplasia (BPD)

**DOI:** 10.3389/fped.2019.00540

**Published:** 2020-01-10

**Authors:** Dandan Chen, Jing Chen, Ningxun Cui, Mingling Cui, Xiaoqian Chen, Xueping Zhu, Xiaoli Zhu

**Affiliations:** ^1^Department of Neonatology, Children's Hospital of Soochow University, Suzhou, China; ^2^Department of Intervention, The First Affiliated Hospital of Soochow University, Suzhou, China

**Keywords:** bronchopulmonary dysplasia (BPD), prognosis, pulmonary function, tidal breathing flow volume loop, respiratory morbidity

## Abstract

**Purpose:** To explore the lung function of bronchopulmonary dysplasia (BPD) in premature infants to guide clinical prevention, early diagnosis and treatment.

**Methods:** Thirty infants with BPD at 4–36 months of corrected gestational age were enrolled and divided into mild BPD and moderate and severe BPD groups. Thirty full-term healthy infants, and 30 non-BPD infants at 4–36 months of corrected gestational age were included as controls. Clinical information, including respiratory infections and re-hospitalization, was compared among these groups. Furthermore, lung function analysis was performed in the infants.

**Results:** The upper respiratory tract infection rate and re-hospitalization rate were significantly higher in the infants with BPD than in the non-BPD infants. The tidal volume/kg, proportion of time to reach peak tidal expiratory flow/total expiratory time, tidal volume exhaled at peak tidal expiratory flow/total tidal volume in BPD group were significantly lower in the BPD group than those in non-BPD group. These values gradually decreased as the severity of BPD increased. The respiratory rate (RR) in BPD group was significantly higher than that in non-BPD group. As the severity of the BPD increased, slope of the descending branch of expiration of tidal breathing flow capacity ring (TBFVL) increased.

**Conclusion:** There is a correlation between the severity of BPD and a poor prognosis of respiratory system. TBFVL can directly reflect the characteristics of Tidal Pulmonary Function in children with different degrees of BPD.

## Introduction

Bronchopulmonary dysplasia (BPD) is the most serious respiratory disorder that is observed in premature infants. The “old” and classic definition of BPD was first proposed by Northway et al. (1). According to this report, BPD is usually observed secondary to severe respiratory distress syndrome, and is characterized by oxygen poisoning, barotrauma, volumetric injury, and sustained oxygen for at least 28 days. Following the implementation of lung protective ventilation strategies, including prenatal glucocorticoids and postnatal pulmonary surfactants, the incidence of classic BPD has significantly reduced. Currently, the term BPD more commonly refers to light BPD (also known as “new” BPD). This definition was adopted by the National Institute of Child Health and Human Development in June of 2001 (2). Light BPD mainly occurs in the immature lung of extremely premature babies without symptoms of respiratory distress syndrome (RDS) or with mild symptoms after birth. In these cases, oxygen delivery is not usually required, or there is only a need for a low concentration of oxygen and oxygen dependence gradually appears during hospitalization. The period of continuous oxygen supply usually exceeds 36 weeks of corrected gestational age (GA). The foremost features of new BPD are delays in lung development and simplified alveolar structure. Following the development of perinatal medicine and technologies of neonatal intensive care, the incidence and survival rate of very low birth weight infants (VLBWI) and extremely low birth weight infants (ELBWI) have increased. However, although there has been a decrease in the rate that other adverse outcomes are observed in preterm infants, the rate of respiratory diseases that are associated with preterm birth has not declined.

Many studies investigating the risk factors of BPD have been reported worldwide. However, few studies have investigated lung function in BPD. In this study, the clinical characteristics, follow-up data, and lung function of premature infants with BPD, as well as non-BPD premature infants and normal lungs of health babies, who were hospitalized in the neonatology department of the Children's Hospital of Soochow University over the same period, were compared and analyzed to explore the respiratory system prognoses. The purpose of this study is to enable early detection of respiratory diseases and pulmonary dysfunction, in order to enable early intervention and treatment, helping to reduce the use of medical resources, improve the disease treatment, and the quality of life of children.

## Methods

### Subjects

Data of premature infants who were diagnosed with BPD and hospitalized in the Neonatology Department of The Children's Hospital of Soochow University between October 1st, 2013 and October 1st, 2017 were collected in this study. The long-term situation of these patients was then recorded between October 1, 2014 and October 2017. Some data of premature infants without BPD and full-term health babies, who were hospitalized over the same period, were collected to serve as controls. This study was approved by The Medical Ethics Committee of The Children's Hospital of Soochow University (ethical examination approval number: 2013LW001).

### Observation

This study is a retrospective analysis. The infants were divided into four groups: a full-term healthy control group, a non-BPD premature infant group, a mild BPD infant group, and a moderate-to-severe BPD infant group. All of the clinical data from the infants [including the conditions of their mothers during pregnancy, the birth situation of the children, the clinical information (manifestation, symptoms, and examinations, diagnosis, and complications), and treatment measures during hospitalization] were collected, compared, and analyzed. Lung function tests and questionnaires were performed at 4–36 months of GA in the premature infant group. These tests were performed at 4–36 months in the full-term infant groups. The lung function and prognosis of the respiratory system of the infants with BPD were analyzed.

### Diagnosis of BPD and Clinical Grading

The diagnosis of BPD was based on the standard of the National Institute of Child Health and Human Development (NICHD). The diagnostic criteria according to the last post-menstrual GA (PMA) (2) were as follows: (1) preterm low birth weight infants, with or without history of mechanical ventilation therapy, oxygen therapy time ≥28 days (PMA ≥ 32 weeks) or 36 weeks of corrected GA (PMA < 32 weeks) who still require oxygen therapy; (2) persistent or progressive respiratory insufficiency; (3) typical X-ray or CT findings of the lungs (such as, both lungs with enhanced texture, reduced permeability, ground glass-like, localized emphysema, or cystic changes); (4) exclusion of congenital heart disease, pneumothorax, pleural effusion and sputum. The clinical grading (2) is based on the aerobic degree of the infants whose GA <32 weeks, the corrected GA of 36 weeks or at discharge, and whose GA ≥ 32 weeks, 56 days of post birth or at the time of discharge were as follows: (i) mild: no oxygen necessary; (ii) moderate: fraction of inspired oxygen (FiO_2_) <30%; iii) severe: FiO2 ≥ 30% and/or continuous positive pressure ventilation or mechanical ventilation.

### Exclusion Criteria

Infants with the following criteria were excluded from the study: (i) a hospitalization time <28 days, (ii) patients with central nervous system and respiratory system malformation, paralysis, or severe complex congenital heart disease (except atrial septal defect, ventricular septal defect, and patent ductus arteriosus), (iii) patients who were transferred to another hospital, died, or showed chromosomal abnormalities or hereditary metabolic diseases within 28 days after birth, due to surgical diseases or other reasons or (iv) patients whose clinical data was incomplete.

### Pulmonary Function Measurement

The measurement was performed by a MasterScreen PAED Lung Function Analyzer (CareFusion, Hoechberg, German) to obtain the flow signals by flow sensors, which were then integrated to obtain the volume data, thus depicting the flow-volume curve under the tidal breathing state. The indices of tidal volume per kg (VT/Kg), respiratory rate (RR), proportion of time to reach peak tidal expiratory flow to total expiratory time (TPTEF/Te), peak volume ratio (VPTEF/Ve), exhalation flow rate (TEF) at the remaining 75% tidal volume (TEF75), TEF50, and TEF25 were recorded.

### Pulmonary Function Analysis

The pulmonary function test was based on the guidelines for tidal breathing and lung function produced by the Respiratory Group Pulmonary Function Cooperative Group of the Chinese Medical Association Pediatrics Branch (3). The test was performed by a designated, trained, and experienced respiratory nurse in a designated and relatively quiet lung function room. The children that were tested did not have respiratory infections in the 2 weeks prior to the analysis, were not taking oral bronchodilator drugs in the week prior to the analysis, and did not show significant abdominal distension 1–2 h after eating. Briefly, the children were sedated with 0.5 ml/kg chloral hydrate, administered orally (if necessary, an enema was used), and placed in the supine position. The necks of the children were then slightly stretched. An appropriate mask was selected to fit tightly around the nose and mouth, to ensure that there was no air leakage. The mask was then connected to the flow sensor. The flow and volume signals of the tidal breathing of the subjects were recorded in real time. Once the breath was stable, 5 consecutive recordings were made. Each recording included at least 20 tidal breathing flow-volume loops. The average value was automatically calculated as the final result by the instrument.

### Statistical Analysis

Collected data were analyzed using SPSS (version 19.0) statistical software (SPSS, Chicago, IL, USA). The measurement data that met the normal distribution are presented as mean ± standard deviation (±s). Comparisons between two groups were examined using *t-*tests. Comparison among groups was analyzed by variance. Non-normal distribution data are expressed as 50% saturation (P50), P25, or P75. The rank sum test was employed for comparison between the two groups. The *K* test was applied for comparison between groups. Count data are presented as (%). *Chi-square test* or Fisher's exact probability method was used for comparison between the two groups (4). *P* < 0.05 was considered statistically significant.

## Results

### Respiratory Infections and Re-hospitalization

The rate of respiratory infection and re-hospitalization in infants under 1 year of age were compared the between the BPD and non-BPD groups. The frequencies of upper respiratory tract infection, pneumonia, wheezing, and re-hospitalization were significantly higher in the BPD group than those in the non-BPD group (*p* < 0.01; Table 1). In addition, the frequency of upper respiratory tract infection, pneumonia, and wheezing were significantly higher in the moderate/severe BPD group than in the mild BPD group. Similarly, these rates were higher in the mild BPD group than in the non-BPD group. Furthermore, the frequency of these conditions was higher in the non-BPD group than in the full term healthy controls (Table 2).

**Table 1:** Lung function analysis in the BPD and non-BPD groups.

**Item**	**BPD (*n =* 30)**	**Non-BPD (*n =* 30)**	**t/U**	***P***
VT/Kg (ml/kg)	6.52 ± 1.70	8.00 ± 1.24	−3.86	<0.001
RR (times/min)	41.35 ± 8.46	33.63 ± 6.48	3.97	<0.001
TPTEF/Te (%)	15.55 (12.08, 17.85)	21.00 (17.95, 22.23)	160.00	<0.001
VPTEF/Ve (%)	19.60 (16.98, 22.33)	22.70 (19.70, 25.80)	271.00	0.008
TEF75 (ml/s)	120.43 ± 37.86	122.59 ± 35.57	−0.22	0.825
TEF50 (ml/s)	90.50 (66.75, 112.50)	107.00 (72.50, 118.00)	357.50	0.439
TEF25 (ml/s)	52.50 (42.25, 75.00)	66.00 (42.50, 80.50)	364.50	0.508

*BPD, bronchopulmonary dysplasia; VT/Kg, tidal volume per kg; RR, respiratory rate; TPTEF/Te, proportion of time to reach peak tidal expiratory flow to total expiratory time; VPTEF/Ve, the fraction of tidal volume exhaled at peak tidal expiratory flow (PTEF) to total tidal volume; TEF75, exhale flow rate at the remaining 75% tidal volume; TEF50, exhale flow rate at the remaining 50% tidal volume; TEF25, exhale flow rate at the remaining 25% tidal volume*.

**Table 2:** Respiratory infections and re-hospitalization in the BPD and non-BPD groups.

**Frequency (time)**	**BPD** **(*n* = 30)**	**Non-BPD** **(*n* = 30)**	**U**	***P***
Up res infection	4.00 (3.75, 5.00)	2.50 (2.00, 3.00)	73.50	<0.001
Pneumonia	2.00 (1.75, 2.25)	1.00 (0.00, 1.00)	78.00	<0.001
Wheezing	2.00 (2.00, 3.00)	0.50 (0.00, 1.00)	60.00	<0.001
Re-hosp	1.50 (1.00, 2.00)	0.00 (0.00, 1.00)	60.00	<0.001

*BPD, bronchopulmonary dysplasia; Up res infection, upper respiratory tract infection; Re-hosp, re-hospitalization*.

### Lung Function Analysis

Compared with the results in the non-BPD group, the lung function parameters, including VT/Kg, TPTEF/Te, and the fraction of tidal volume exhaled at peak tidal expiratory flow to total tidal volume (VPTEF/Ve), were lower in the BPD group (*p* < 0.05). However, RR was higher in the non-BPD group than in the BPD group (*p* < 0.05). There was no significant difference in the TEF75, TEF50, or TEF25 between the two groups (*p* > 0.05; Table 3). The lung function analysis among the four groups indicated that the TPTEF/Te, VPTEF/Ve, and VT/Kg in the moderate and severe BPD group were lower than those in the mild BPD group (*p* < 0.05). These same parameters were lower in the mild BPD group than in the non-BPD group (*p* < 0.05). Furthermore, these parameters were lower in the non-BPD group than in the full-term healthy controls (*p* < 0.05; Table 4).

**Table 3:** Respiratory infections and re-hospitalization in the four groups.

**Frequency (time)**	**Full-term** **(*n =* 30)**	**Non-BPD** **(*n =* 30)**	**Mild-BPD** **(*n =* 15)**	**M/S BPD** **(*n =* 15)**	**H**	***P***
Up res infection	1.00 (1.00, 2.00)^bdf^	2.50 (2.00, 3.00)^df^	3.50 (3.00, 4.00)^e^	5.00 (4.00, 5.00)	62.12	<0.001
Pneumonia	0.00 (0.00, 1.00)^df^	1.00 (0.00, 1.00)^de^	1.50 (1.00, 2.000)^e^	2.00 (2.00, 3.00)	52.29	<0.001
Wheezing	0.00 (0.00, 0.00)^adf^	0.50 (0.00, 1.00)^de^	2.00 (2.00, 3.000)^e^	3.00 (2.00, 3.00)	57.48	<0.001
Re-hosp	0.00 (0.00, 1.00)^df^	0.00 (0.00, 1.00)^de^	1.00 (1.00, 2.00)^e^	2.00 (1.00, 2.00)	62.14	<0.001

*BPD, bronchopulmonary dysplasia; M/S BPD, moderate and severe BPD group; Up res infection, upper respiratory tract infection; Re-hosp, re-hospitalization; a, the frequency is higher in the Full-term group than in the Non-BPD group, P < 0.05; b, the frequency is higher in the Full-term group than in the Non-BPD group, P < 0.001; d, the frequency is higher in the Full-term group than in the Mild-BPD group, P < 0.001; e, the frequency is higher in the Full-term group than in the M/S BPD group, P < 0.05; f, the frequency is higher in the Full-term group than in the M/S BPD group; P < 0.001*.

**Table 4:** Lung function analysis in four groups.

**Item**	**Full-term** **(*n =* 30)**	**Non-BPD** **(*n =* 30)**	**Mild BPD** **(*n =* 15)**	**M/S BPD** **(*n =* 15)**	**H**	***P***
VT/Kg (ml/kg)	8.45 (6.35, 9.43)^ace^	8.20 (6.98, 8.93)^cf^	6.35 (5.75, 7.60)^e^	6.00 (5.20, 7.60)	14.78	0.002
RR (times/min)	34.90 (27.08, 41.60)^f^	34.00 (28.15, 38.95)^f^	42.70 (33.95, 47.00)	45.00 (40.00, 47.00)	20.19	<0.001
TPTEF/Te (%)	26.40 (23.53, 27.80)^bdf^	21.00 (17.95, 22.23)^cf^	15.55 (12.08, 17.85)^f^	12.10 (11.70, 17.00)	53.43	<0.001
VPTEF/Ve (%)	28.85 (26.60, 30.70)^bdf^	22.70 (19.70, 25.80)^cf^	19.60 (16.98, 22.33)^f^	17.00 (15.10, 19.30)	51.06	<0.001
TEF75 (ml/s)	113.50 (86.75, 137.75)	117.70 (97.50, 137.50)	120.0 (93.50, 141.00)	121.50 (98.75, 162.50)	0.46	0.928
TEF50 (ml/s)	109.50 (81.75, 130.00)	90.50 (66.75, 112.50)	107.0 (72.50, 118.00)	101.00 (73.50, 120.00)	2.86	0.413
TEF25 (ml/s)	79.50 (60.50, 87.00)^ace^	52.50 (42.25, 75.00)	66.00 (42.50, 80.50)	62.50 (40.00, 79.50)	7.37	0.041

*BPD, bronchopulmonary dysplasia; VT/Kg, tidal volume per kg; RR, respiratory rate; TPTEF/Te, proportion of time to reach peak tidal expiratory flow to total expiratory time; VPTEF/Ve, the fraction of tidal volume exhaled at peak tidal expiratory flow (PTEF) to total tidal volume; TEF75, exhale flow rate at the remaining 75% tidal volume; TEF50, exhale flow rate at the remaining 50% tidal volume; TEF25, exhale flow rate at the remaining 25% tidal volume. ^a,b^P < 0.05 and 0.001, respectively, compared to the non-BPD group results; ^c,d^P < 0.05 and 0.001, respectively, compared to the mild BPD group results; ^e,f^P < 0.05 and 0.001, respectively, compared to the moderate and severe BPD group results*.

### Lung Function Analysis in Different Age Groups

The lung function analysis in the different age groups indicated that the TEF75 and TEF50 in the 12–24 and 24–36 month groups were higher than those in the 6–12 and < 6 month groups (*p* < 0.05). The TEF75 and TEF50 in the 6–12 months below group were higher than those of the < 6-month group (*P* < 0.05). The TEF25 gradually increased with age (Table 5).

**Table 5:** Lung function analysis in different age groups.

**Item**	**<6 M** **(*n =* 28)**	**6–12 M** **(*n =* 31)**	**12–24 M** **(*n =* 21)**	**24–36 M** **(*n =* 10)**	***F***	***P***
VT/Kg (ml/kg)	7.24 ± 1.17	7.61 ± 2.11	8.15 ± 2.11	8.51 ± 2.11	1.03	0.382
RR (time/min)	40.80 ± 9.80^c^	37.15 ± 8.32^b^	37.05 ± 8.53	31.49 ± 7.86	4.56	0.005
TPTEF/Te (%)	20.53 ± 5.15	20.45 ± 6.39	20.76 ± 5.81	18.58 ± 7.55	1.24	0.299
VPTEF/Ve (%)	23.65 ± 4.91	23.70 ± 5.35	23.11 ± 5.36	22.21 ± 7.69	0.68	0.565
TEF75 (ml/s)	100.48 ± 26.28^ace^	114.33 ± 25.88^be^	138.65 ± 41.51	157.70 ± 41.50	10.47	<0.001
TEF50 (ml/s)	80.00 ± 28.93^ace^	96.43 ± 25.44^be^	119.15 ± 31.06	141.00 ± 47.56	12.25	<0.001
TEF25 (ml/s)	53.37 ± 20.62^ace^	63.90 ± 22.68^be^	79.50 ± 22.50^d^	100.80 ± 41.41	10.89	<0.001

*M, months; VT/Kg, tidal volume per kg; RR, respiratory rate; TPTEF/Te, proportion of time to reach peak tidal expiratory flow to total expiratory time; VPTEF/Ve, the fraction of tidal volume exhaled at peak tidal expiratory flow (PTEF) to total tidal volume; TEF75, exhale flow rate at the remaining 75% tidal volume; TEF50, exhale flow rate at the remaining 50% tidal volume; TEF25, exhale flow rate at the remaining 25% tidal volume. ^a^P < 0.05, respectively, compared to the 6–12 month group results; ^b,c^P < 0.05 and 0.001, respectively, compared to the 12–24 month group results; ^d,e^P < 0.05 and 0.001, respectively, compared to the 24–36 month group results*.

### Tidal Breathing Flow Capacity Ring (TBFVL) Status

**Figure 1:**
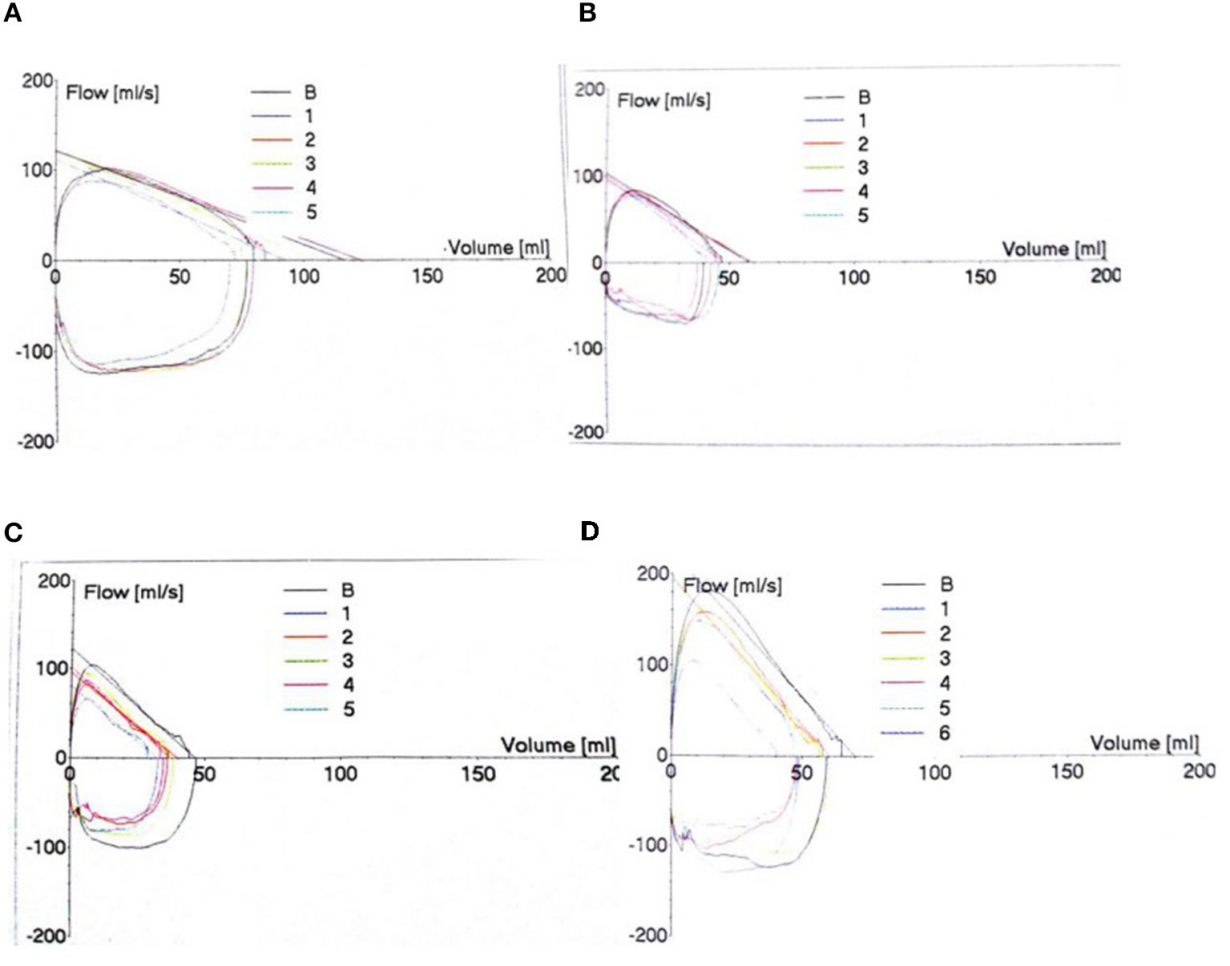
Tidal Breathing Flow Capacity Ring (TBFVL) Status. The changes in TBFVL were recorded in the non-BPD **(A)**, mild **(B)**, moderate **(C)** and severe **(D)** BPD groups.

Taking the 4 months of corrected gestational age as an example, the inspiratory phase was relatively smooth, the peak of exhalation was advanced, and the expiratory phase was steeper in the BPD group than in the Non-BPD groups. The more severe the BPD, the steeper the descending branch and the more severe of the slope (Figure 1).

## Discussion

BPD is the most common pulmonary complication in preterm infants. This condition is associated with a variety of risk factors and multiple long-term effects (5). BPD can reduce lung function (6), physical activity, and cardiovascular function (7). These effects can be sustained into adulthood (8). Therefore, BPD has a serious impact on the public health system, due to the burden it places on medical resources. In the past decade, researchers have described the long-term sequelae of premature survivors. These studies have mainly focused on the lungs and the nervous system. However, in this field, few studies have been performed in China. Previous studies have indicated that infants with BPD have an increased risk of respiratory infections, higher rates of re-hospitalization (9) and are more prone to wheezing and recurrent wheezing (10). Abnormalities in persistent lung function are more common in infancy, and the extent of the abnormalities depends on the severity of BPD (11). The data in this study suggest that the infants with BPD had more respiratory infections, pneumonia, wheezing and a higher frequency of re-hospitalization than those in the non-BPD premature group. There was a correlation between the BPD severity and the number of infections, which is consistent with the above findings.

BPD-induced pulmonary dysfunction is a restrictive or obstructive disorder syndrome that changes over time. The restrictive dysfunction is more severe clinically. However, the obstructive dysfunction is more common (12). Due to alveolar and pulmonary microvascular developmental delay in BPD infants (13), the number of alveoli was lower than in non-BPD infants. Furthermore, BPD infants showed simplified alveolar structure, mild fibrosis of airway and surrounding tissue, and microvascular dysplasia, leading to increased pulmonary vascular resistance and pulmonary vascular remodeling (14). Colin et al. (15) reported that the high elasticity of the chest wall of the newborn resulted in a decrease in transpulmonary pressure and a reduction in the lung capacity during expiration and closed airway, resulting in a decreased flow rate. Because the elasticity of the chest wall was higher in premature infants, the decreased expiratory flow rate was more pronounced in this group. Friedrich et al. (16) believed that, due to the immature lung development in premature infants, even if the lung capacity is normal after birth, the respiratory flow rate would still be decreased. This suggests that the long-term development of the airway is delayed. Our study found that premature infants with BPD had small airway obstructions. The more severe the BPD disease, the more obvious the small airway obstruction was. Thus, in severe cases of BPD, oxygen supply would be more reliant on the increased respiratory rate for compensation to maintain normal ventilation function. The lung volume flow rate increased significantly after 1 year of age. However, the small airway obstruction was still observed. This suggests that the development of premature infants with lung dysplasia is dominated by the lung capacity development, while their airway development is delayed. All of these results are consistent with the above findings.

The tidal breathing flow volume loop can reflect the presence or absence of an obstruction in the airway, the degree and location of the obstruction, and the ventilatory function. This measurement assists in diagnosing the of nature of the pulmonary disease and the degree of lung damage, providing an objective indicator for clinical evaluation. Tidal pulmonary function is a new technology that reflects the lung function of infants and young children. The advantage of this method is that it is a safe, non-invasive, sensitive, and accurate reflection of changes in lung capacity and ventilation function. Our analysis indicates that the inspiratory phase of the BPD premature infants was relatively smooth and nearly a half elliptical shape. The exhalation curve, however, was not smooth. The peak of exhalation occurred sooner in BPD premature infants. The expiratory decline was steep. The more severe the BPD, the larger the slope of the descending branch. These findings support the work of Wei et al. (17).

This study was a small-sample study in a single center and external cohort verification was not performed. Because of time constraints, some parents did not aware the importance of early intervention, some patients with severe BPD died after they were discharged or transferred to other hospitals, resulting in fewer follow-up cases than expected. The larger the follow-up age span, the more discrete the observation indicators. In addition, we did not carry out dynamic follow-up assessment, thus, the results do not reflect the trend of dynamic alteration of the indices along with the age increase. Therefore, it would be valuable to perform further studies with a larger sample size, longer follow-up period, dynamic follow-up, and fully internal and external cohort verification.

In summary, the etiology of BPD is complex and diverse. BPD can cause abnormal lung function and seriously affect the prognosis. Therefore, clinicians should develop a preventive strategy for BPD, provide prenatal guidance, avoid the risk factors for BPD as much as possible, and establish follow-up management after discharge, systemically improving the long-term prognosis of children with BPD.

## Data Availability Statement

The raw data supporting the conclusions of this manuscript will be made available by the authors, without undue reservation, to any qualified researcher.

## Ethics Statement

The studies involving human participants were reviewed and approved by the Medical Ethics Committee of The Children's Hospital of Soochow University (ethical examination approval number: 2013LW001). Written informed consent to participate in this study was provided by the participants' legal guardian/next of kin.

## Author Contributions

DC participated in study design and protocol development and writing of the manuscript. JC carried out the data analysis and interpretation of data. NC, MC, and XC participated in clinical data collection. XuZ participated in data analysis, interpretation of data and writing of the manuscript. XiZ participated in the design of the study and coordination. All authors read and approved the final manuscript.

### Conflict of Interest

The authors declare that the research was conducted in the absence of any commercial or financial relationships that could be construed as a potential conflict of interest.

## Abbreviations

AUC: area under the curve
BPD: bronchopulmonary dysplasia
BTT: blood transfusion times
BW: body weight
CI: confidence interval
ELBWI: extremely low birth weight infants
FiO_2_: fraction of inspired oxygen
GA: gestational age
nCPAP: nasal continuous positive airway pressure
NEC: necrotizing enterocolitis
NRDS: neonatal respiratory distress syndrome
OIT: oxygen inhalation time
P50: 50% saturation
PCO_2_: pressure carbon dioxide
PDA: patent ductus arteriosus
PEEP: positive end expiratory pressure
PIP: peak inspiratory pressure
PMA: post-menstrual gestational age
ROC: receiver operating characteristic
RF-II: type II respiratory failure
RR: respiratory rate
sd: standard deviation
TBFVL: tidal breathing flow capacity ring
TEF75: exhale flow rate at the remaining 75% tidal volume
TPTEF/Te: proportion of time to reach peak tidal expiratory flow to total expiratory time
VLBWI: very low birth weight infants
VPTEF/Ve: the fraction of tidal volume exhaled at peak tidal expiratory flow (PTEF) to total tidal volume
VT/Kg: tidal volume per kg.
